# Heavy migration of flood affectees in Pakistan and increased risk of autism spectrum disorder in children: a call to action

**DOI:** 10.1097/JS9.0000000000000136

**Published:** 2023-03-03

**Authors:** Syeda T. Rehan, Mariam Ahmed, Arooba N. Bukhsh, Kinza Bari, Hassan ul Hussain, Irfan Ullah, Muhammad Sohaib Asghar

**Affiliations:** a Dow University of Health Sciences; b Jinnah Sindh Medical University; c Karachi Medical and Dental College, Karachi; d Kabir Medical College, Gandhara University; e Institute of Public Health and Social Sciences (IPH&SS), Khyber Medical University, Peshawar; f Dow University of Health Sciences-Ojha Campus, Karachi, Pakistan

**Keywords:** autism, flood, mental health, migrants, relief

## Abstract

Autism spectrum disorder (ASD) refers to various neurodevelopmental disabilities generally seen in kids. Pakistan, being vulnerable to natural disasters, faced one of the most devastating floods in July 2022 due to which many individuals were displaced. This not only affected the mental health of growing children but also the developing fetus of migrant mothers. This report establishes the link between the aftereffects of migration due to floods on children particularly associated with ASD in Pakistan. Flood affected families are devoid of basic necessities and are under a lot of psychological stress. On the other hand, Extensive treatment for autism is complicated, expensive, and provided in proper settings only which is not easily accessible to migrants. Considering all these factors, there are chances that ASD will be more prevalent in future generations of these migrants. Our study calls on respective authorities to take timely action for this growing concern.

Highlights

- Autism spectrum disorder affects the mental health of growing children.
- This report establishes the link between the aftereffects of migration due to floods on children particularly associated with autism spectrum disorder in Pakistan.
- Flood affected families are devoid of basic necessities and are under a lot of psychological stress. Our study calls on respective authorities to take timely action for this growing concern.

Autism spectrum disorder (ASD) is a neurodevelopmental disability that often manifests before the age of three and is marked by persistent delays in social interaction^1^. It is a psychiatric illness characterized by symptoms of impaired language and cognition, restricted social interaction, and repetitive and stereotyped patterns of behaviors or interests^2^. According to current estimates, ASD affects up to 1 in 36 children worldwide, and 75% of those children also have one or more co-occurring mental health conditions, such as attention-deficit hyperactivity disorder, bipolar disorder, depression, or anxiety^3^. It has been observed that several hereditary variables, such as parental age, maternal stress, smoking, exposure to certain drugs, and improper brain development during pregnancy may raise the chance of ASD in developing children^4^. Moreover, low birth weight, metal exposure, and a sibling with autism also account for a high risk of developing ASD^5^.

Research has suggested that migrant parents are more prone to having ASD in their children than non-migrants^4^. A study by Mughal *et al.* 6, concluded that children of immigrants exhibit unpleasant behavior when exposed to geographic differences because they have weaker or less adaptive abilities. Maternal migration during pregnancy increases the risk of ASD in kids^4^,^7^. Migration is a challenging and diverse process that involves a variety of elements. Migration-associated stress is associated with poor physical and mental health in migrants and their offspring^7^. Migration exposes the mothers to multiple infections, and physical and mental stress and may contribute to the neuropsychological impairment of the fetus^7^. Children of migrants are at increased risk of autism and exhibit more severe disease as they frequently fail to receive timely care due to the hindrance in receiving health care facilities. Due to socioeconomic limitations, children of migrants have fewer opportunities of receiving an early diagnosis, social deprivation caused by lack of access to educational facilities, and exposure to language and cultural differences make it difficult for them to communicate and associate with the local health care system; and hinder their mental development^8^.

Pakistan has been a frequent victim of floods; however, the magnitude of destruction and devastation experienced in the year 2022 has been incomparable. Over 33 million people were affected, of which 6.4 million lost homes and all their belongings. Approximately 300,000 houses have been demolished, while the flooding has damaged over 650,000^9^. Distress arising from floods exposes flood victims to higher chances of developing psychiatric problems^10^.

A 2020 study stated that the Pakistan Autism Society has calculated that there are about 350,000 kids in Pakistan who have been diagnosed with ASD^11^. Recent floods have left almost 634,000 affectees to migrate from their home regions and reside in shelters and camps^12^. Among the flood victims were nearly 130,000 pregnant women with 42,000 due to give birth amidst the peak of the crisis. Most women of reproductive age are malnourished and have to deal with problems like water-borne diseases which include cholera, malaria, and dysentery^13^. Pregnant women who are in urgent need of humanitarian assistance have to wait for hours to receive any sort of medical attention; this drastically increases their risk of developing stress and other physical illnesses^13^. These stats show a large-scale migration hence the greater chances of ASD in progeny.

Extensive treatment for autism is complicated, expensive, and only offered in special settings; therefore, it is challenging for migrant families to access such care^14^. In certain cases, care centers are not available within their locality. In these situations, limited transportation and money impose serious challenges for migrants^15^. Considering these factors, we think that ASD will be more prevalent in future generations of these migrants, and already existing kids who had poor school performance or lack of concentration are at higher risk of developing ASD, while children already having a diagnosis of ASD will have a less positive response to treatments.

The government should launch campaigns and work with psychologists to assess the mental wellbeing of migrants. Those who are deeply traumatized should be provided with immediate care and therapy. Migrants should be provided with shelter and all the necessities, including food, clean water, sanitation to have a sense of security and safety, so they do not feel vulnerable. In addition, victims’ families should be empowered through education and employment opportunities to have a routine and sense of stability. Small-scale community training programs should be organized to teach basic disaster response skills so that the migrants are more aware of their surroundings and feel mentally prepared if they face the same situation. Psychological assessment of newborn kids of migrants in the first few years of their life with long-term follow-up is also crucial so that children with special psychological needs are recognized and catered to with special care from the beginning. This will improve their quality of life and reduce the chances of their condition getting worse.

## Ethical approval

Not applicable.

## Sources of funding

None.

## Author contribution

S.T.R. and M.A.: conceived the idea. A.N.B., K.B., H.u.H., I.A., and M.A.: collected the data. S.T.R. and M.S.A.: analyzed and interpreted the data. I.U. and S.T.R. did write up of the manuscript. M.S.A.: reviewed and revised the manuscript for intellectual content critically. All authors approved the final version of the manuscript.

## Conflicts of interest disclosure

The authors declare that they have no financial conflict of interest with regard to the content of this report.

## Research registration unique identifying number (UIN)

None.

## Guarantor

Muhammad Sohaib Asghar.

## Provenance and peer review

Externally peer reviewed, not commissioned.
